# Bioaccumulation of Macronutrients in Edible Mushrooms in Various Habitat Conditions of NW Poland—Role in the Human Diet

**DOI:** 10.3390/ijerph18168881

**Published:** 2021-08-23

**Authors:** Ryszard Malinowski, Zofia Sotek, Małgorzata Stasińska, Katarzyna Malinowska, Patrycja Radke, Alicja Malinowska

**Affiliations:** 1 Department of Environmental Management, West Pomeranian University of Technology in Szczecin, Słowackiego 17 Street, 71-434 Szczecin, Poland; 2 Institute of Marine and Environmental Sciences, University of Szczecin, Adama Mickiewicza 16 Street, 70-383 Szczecin, Poland; zofia.sotek@usz.edu.pl (Z.S.); malgorzata.stasinska@usz.edu.pl (M.S.); 3 Department of Bioengineering, West Pomeranian University of Technology in Szczecin, Słowackiego 17 Street, 71-434 Szczecin, Poland; katarzyna.malinowska@zut.edu.pl; 4 Institute of Biology, University of Szczecin, Wąska 12 Street, 71-899 Szczecin, Poland; patrycjaradke7@gmail.com; 5 Faculty of Medicine and Dentistry, Major Medicine, Pomeranian Medical University in Szczecin, Rybacka 1 Street, 70-204 Szczecin, Poland; Ala.95@op.pl

**Keywords:** fungi, macroelements, properties of soil, *Boletus edulis*, *Leccinum scabrum*, *Imleria badia*

## Abstract

Recently, the interest in mushroom consumption has been growing, since their taste and low calorific value are appreciated, but also due to their nutritional value. In determining the usefulness of mushrooms in the human diet, it is important to consider the conditions of their occurrence to perform the assessment of bioaccumulation of minerals. The aim of the study was: (a) to determine the content of selected macronutrients (P, K, Ca, Mg, Na) in fruiting bodies of *Boletus edulis*, *Imleria badia*, *Leccinum scabrum* and the soils, (b) to determine their bioaccumulation potential taking into account the habitat conditions, and (c) an attempt to estimate their role in covering the requirement for macronutrients of the human organism. The research material was obtained in the NW of Poland: Uznam and Wolin, the Drawa Plain and the Ińsko Lakeland. In the soil, we determined the content of organic matter, pH, salinity and the content of absorbable and general forms of macronutrients. The content of macronutrients in mushrooms was also determined. Chemical analyses were performed using the generally accepted test methods. The study showed that in NW Poland, *B. edulis* grew on the acidic soils of Arenosols, and *I. badia* and *L. scabrum* grew on Podzols. The uptake of K, Mg and Ca by the tested mushrooms was positively, and P and Na negatively correlated with the content of these elements in the soil. The acidity of the soil affected the uptake of K and Mg by mushrooms. There was no effect of the amount of organic matter in the soil noticed on the content of macronutrients (except sodium) in mushrooms. Among the studied macronutrients, none of the mushrooms accumulated Ca, while P and K were generally accumulated in the highest amounts, regardless of the species. Each of the other elements was usually accumulated at a similar level in the fruiting bodies of the species we studied. The exception was *I. badia*, which accumulated higher amounts of Mg compared to *B. edulis* and *L. scabrum*. Mushrooms can enrich the diet with some macronutrients, especially in P and K.

## 1. Introduction

Wild edible mushrooms in many countries, especially in central and eastern Europe in autumn, when they appear more often, are a frequent element in the human diet. Recently, the interest in their consumption has been increasing, not only because of the taste, but also due to their nutritional value [1,2,3]. The main mass of mushroom fruiting bodies is water, on average 90% by weight. On the other hand, the dry matter of mushrooms has a diversified composition. Depending on the species, they contain carbohydrates in the range from about 3 to 42% DM, protein from 4 to 44 (max 57.3)% DM and lipids from 2 to 6% DM [4,5]. This differentiation occurs even within the same genus, e.g., *Boletus* spp. contain in dry matter 21.6 to 25.8% crude protein, 61.7–75% carbohydrates, 3.0–5.8% lipids and 5.7–8.2% ash [6,7]. Mushrooms contain more and qualitatively better nutritional protein than most plants [4,8,9]. Moreover, they are characterized by a low caloric value, generally not exceeding 200 kJ/100 g of dry matter. They also contain many vitamins from the C and B group (niacin, folic acid), small amounts of vitamin D and several polyphenols and carotenoid [10,11,12]. Like plants, they contain significant amounts of macroelements, especially a lot of phosphorus and potassium, slightly less magnesium, and small amounts of sodium and calcium [4,13,14,15]. However, the degree of bioavailability of these elements from consumed mushrooms is as of yet unknown [4,16].

The growth of mushrooms and their uptake of macroelements is closely related to the chemical and physical properties of soil, including organic matter, and in case of mycorrhizal mushrooms, also to the symbiosis with plant roots [17,18,19]. Due to the specific structure of the mycelium—the exposed surface of vegetative cells and the large surface of hyphae—mushrooms easily absorb minerals even at small amounts in the soil, which often results in a much higher concentration of minerals in fruiting bodies compared to the substrate [20,21,22,23]. Mushrooms also have the ability to absorb elements from dust deposited on fruiting bodies [8]. The excess of accumulated macronutrients (e.g., P and N) is used by mycorrhizal mushrooms to exchange with plants for the products of photosynthesis [24,25,26].

Poland is one of the leading producing countries for mushrooms. In 2017, 7302 tons of mushrooms were collected here, and in 2018, 3261 tons, including 2057 tons of bay boletes and 248 tons of boletuses, while in 2019, 5912 tons were collected [27]. The differentiation in the purchase of mushrooms in particular years was related to the weather conditions. However, regarding the popularity of mushroom picking in local communities, these figures do not reflect the actual amount of mushrooms actually consumed. Therefore, due to the ability to accumulate macronutrients, mushrooms can—at least when consumed often—constitute a significant supplement to the human diet with these elements. In Poland, the most commonly harvested wild mushrooms include the species which are the subject of our research: *Boletus edulis*, *Imleria badia* and *Leccinum scabrum*. Previous studies on the usefulness of these species in covering the demand for minerals necessary for the proper development and functioning of the organism did not take into account the conditions of their accumulation by fruiting bodies of mushrooms from NW Poland. Therefore, our research focused on this region. The aim of the study was: (a) to determine the content of selected macronutrients (P, K, Ca, Mg, Na) in fruiting bodies of *B. edulis*, *I. badia*, *L. scabrum* and the soils; (b) to determine their bioaccumulation potential taking into account the habitat conditions; and (c) an attempt to estimate their role in covering the requirement for macronutrients of the human organism.

## 2. Materials and Methods

### 2.1. Study Area

The mushrooms were collected in three physiographic regions of NW Poland: Uznam and Wolin, the Drawsko Plain and the Ińsko Lakeland [28]. Uznam and Wolin are the islands separating the Szczecin Lagoon from the Pomeranian Bay. Mushrooms for research were collected in the north-eastern part of the Wolin island. The low ridges of shore dunes covered by dry pine forests create the dominant topography in that region. The Drawsko Plain is an extensive outwash plain drained by Drawa river and its tributaries. The quite monotonous landscape of this region is diversified by numerous lakes, mid-forest pools and peat bogs. The Drawsko Plain is covered mainly by pine forests. The Ińsko Lakeland is hilly morainic upland intersected by numerous glacial troughs. Apart from the moraine hills and ravines, the region has numerous post-glacial lakes and pools, and vast water-logged area. In this region, the dense forests are present mostly in the zone of terminal moraine.

### 2.2. Fungal and Soil Materials

Three species of the most commonly picked wild-growing edible mushrooms in Poland were selected for the study: *Boletus edulis*, *Imleria badia* and *Leccinum scabrum*. The locations of material collection were in the distance from communication routes, and there were no other sources of environmental pollution in these areas as well. During the sampling, attention was paid to make sure that the fruiting bodies of the same species are collected in different places, are fully developed and are not attacked by insects, snails and molds. From each of the three studied regions, 3–7 pooled samples of each species were collected, with each pooled sample consisting of 5 fruiting bodies. The mushrooms were cleaned of sand and bedding, and after being transported to the laboratory, were dried in an electric dryer at 40 °C for 48 h. After drying, the whole fruiting bodies were milled to a powder in a mortar. The average dry weight of the pooled sample of *Boletus edulis* from Uznam and Wolin, Drawsko Plain, and Ińsko Lakeland averaged 10.63 g, 13.50 g, 8.60 g, respectively; of *I. badia* 7.25 g, 7.61 g, 7.55 g, respectively; and *L. scrabum* 7.96 g, 7.59 g, 7.78 g, respectively. The taxonomic identification of mushrooms was made according to Knudsen and Vesterholt [29], using standard methods of macroscopic mushroom testing, and the names of the species were given according to the Index Fungorum database (http://www.indexfungorum.org/ (accessed on 15 June 2021)).

In each fruiting bodies picking location, the soil substrate was also collected from a depth of 0–20 cm for testing. Within the sample, the surface organic level (0–5(10) cm, about 0.5 kg; decomposed forest litter) and the mineral level below (5(10)–20 cm, about 0.5 kg) were collected.

In the soil material the following determinations were made: loss on ignition (organic matter—OM) was determined by burning soil samples in a muffle furnace at the 550 °C; pH in 1 mol dm^−3^ KCl was determined potentiometrically; salinity was determined conductometrically.

Content of available P, Mg, K and Ca was determined by extracting in 0.5 mol∙dm^−3^ HCl; content of total forms in soil macroelements was determined after mineralization in HNO_3_ and HClO_4_ in a ratio of soil 1:1. The content of K, Na and Ca was measured with the atomic emission spectrometry, whereas Mg content was determined by flame atomic absorption spectroscopy using iCE 3000 Series. The content of available and total P was determined by spectrophotometric molybdenum blue method (690 nm wavelength) using spectrophotometer Marcel MEDIA™ [30]. The limits of were detection (mg∙kg^−1^): Ca 0.004; Mg 0.002; K 0.001 and Na 0.004. Assessments of the accuracy and precision of the analytical methods and procedures used were determined using certified reference material: CRM036–050 Loamy Sand 4 (CRM 036-050 produced by Resource Technology Corporation, USA and UK). The effectiveness of the process has been validated with 90–95% efficiency. The results shown are the average of three measurements, working standards made from Merck standards with a concentration of 1000 mg∙dm^−3^.

The content of elements in mushrooms were determined after mineralization of 1 g of mushroom dry weight: Mg, P, K, Na and Ca were measured after wet mineralization in H_2_SO_4_ and HClO_4_. The content of K, Na and Ca was measured with the atomic emission spectrometry, Mg content with the flame atomic absorption spectroscopy. P was assessed by the colorimetric method. The efficiency of the process was validated with 90–95% success using certified reference materials, namely, tea leaves (INCT-TL-1) and a mixture of Polish herbs (INCT-MPH-2), both produced by the Institute of Nuclear Chemistry and Technology, Warsaw, Poland. All tests were performed in three replications.

The coefficient of bioconcentration of macronutrients was calculated using relation:

- BCF = Cm/Cs
- BCF—coefficient of bioconcentration
- Cm—is the concentration of macronutrient in mushroom
- Cs—is the concentration of macronutrient in mushroom substrate (soil)

### 2.3. Statistical Analysis

Statistical analysis of the obtained results of soil chemical properties was performed using Statistica 12.5 (StatSoft Polska, Cracow, Poland).

Statistical significance of differences between means was determined by testing normality of distribution in each group and homogeneity of variance in all groups, followed by ANOVA with Tukey’s post hoc test. The significance was set at *p* < 0.05.

The multidimensional analysis was carried out using the analysis of the main components (PCA). The data were scaled during pre-processing automatically. The obtained results were subjected to agglomerative cluster analysis and classified into groups in a hierarchical arrangement by Ward’s method.

## 3. Results and Discussion

### 3.1. Soil Properties and Macronutrients Concentrations

*B. edulis* were found in Arenosols soils, whereas *I. badia* and *L. scabrum* in Podzols soils [31]. Arenosols soils were characterized by a well-developed organic level with an average organic matter content of 62%, which was deposited at the humus level made of clay sand, with an average organic matter content of 9.74%. Podzols soils had a fleshy organic level with a clear division into the raw material, butvin and epihumus sub-levels, with an average organic matter content of 71.18% under bay boletes, and 64.50% under birch boletes. The humus levels located below the organic levels were rich in organic matter (from 21.37% under bay boletes to 35.22% under birch boletes). The studied Podzols and Arenosols were characterized by the typical features for these types of soil [32,33,34,35,36].

The organic and humus levels in both Arenosols and Podzols were strongly acidic, which is typical for these types of soil. The highest pH values were found in Arenosols under *B. edulis*, and the lowest in Podzols under *I. badia* (Table 1). These soils were characterized by a low electrolytic conductivity—from 109.89 under *B. edulis* to 159.81 µS/cm under *L. scabrum*. Mleczek [37] found that the salinity of soils under *B. edulis*, *I. badia*, *L. scabrum* and other mushrooms stays in the range of 22 to 144 mS/m.

The organic level of Arenosols under *B. edulis* was moderately rich in available K and Mg and very rich in available P [38]. Significant differences were found in the content of K and Mg in the individual research points (from low to very high content of available K and from medium to high content of available Mg). Podzols organic levels were poor in available K and Mg, and very rich in available P [38]. The organic level of Arenosols under *B. edulis* was the most abundant in available K, Mg and P. There were no significant differences in the available Ca content, which accounted for about 80% of its total forms. In the total content of K and P, a high proportion of available forms of K: 69–82% and P: 51–69%, and a lower share of available Mg 28–39% were also found.

In contrast to the organic levels, the sandy mineral levels of Arenozols and Podzols are very poor in nutrients. The studied soils under *B. edulis* were richer in Ca and Mg but poorer in Na and K (Table 2), than the soils under *B. edulis* from other regions of Poland [39]. On the other hand, the content of macroelements in soils under *I. badia* was within the ranges given by Malinowska et al. [40].

### 3.2. Macronutrient Concentrations in Mushrooms

Mushrooms are responsible for the digestion of cellulose, chitin and all dead organic matter. They absorb macro- and microelements from the decomposing organic matter, and in certain amounts pass them further to plants (to the tree root system) in exchange for sugars [24,25]. The mycelium supplies plants with hardly available N and P anions in exchange for sugars, which the mycelium cannot produce itself, due to the lack of photosynthesis process in its cells [26].

#### 3.2.1. Phosphorus

Our research showed that *B. edulis*, *I. badia* and *L. scabrum* did not differ significantly in the P content, containing on average: 8.92 g/kg DM, 8.19 g/kg DM, and 7.70 g/kg DM, respectively (Table 3). Vogt and Edmonds [41], Nikkarinen and Mertanen [18], and Rudawska and Leski [42] also found no differences in the content of this element. In various regions of Poland, its average concentration in the examined species of mushrooms ranged from 1.4–10.0 g/kg DM [42,43,44,45]. In mushrooms growing in Germany, its typical content is 5–10 g/kg DM [4], while in Finland, the content is an average of 4–6.3 g/kg DM [18]; a much higher concentration was recorded in Japan, from 43–69 g/kg DM [46], whereas small levels were noted in Turkish *L. scabrum*, with a mean value of 3.22 g/kg DM [47]. The mushrooms we analyzed contained amounts of phosphorus that were within the range found in cultivated mushrooms: *Agaricus bisporus* and *Pleurotus ostreatus* [48,49].

Perez-Moreno and Read [24], Entry et al. [50], and Andersson et al. [51] showed that mycelium obtains large amounts of phosphorus from the substrate and supplies it to plants, as well as accumulates it itself in large amounts. The intake of P to the mycelium is 10 to 50 times higher than the levels of this element accumulated in the substrate [42]. In the fungi, we also observed bioconcentration of P, although to a much lesser extent, and the BCF bioconcentration coefficient was similar regardless of the species (Table 4). Bioconcentration of phosphorus in mushrooms was found by Kojta et al. [44], Chudzynski et al. [52], Falandysz et al. [53], Chudzyński and Falandysz [15] and Bučinová et al. [54]. Bučinová et al. [54] found a significantly higher bioconcentration factor for phosphorus in fungi with soil mineral levels than organic levels. A similar relationship was found in our own research. The ability of mushrooms to obtain phosphorus from organic compounds results from the presence of phosphatase produced in their cells [55,56]. Phosphorus is one of the main elements in mushrooms, and it is generally present in lower amounts than potassium [16,18,42,44]. However, in the mushroom fruiting bodies we examined, phosphorus was found in a higher concentration than the other macroelements (Table 3).

Due to the significant potential of phosphorus accumulation, mushrooms can be an important source of this element in the human diet. The recommended daily allowance (RDA) of P according to the Institute of Nutrition and Food [57] ranges from 700 to 1250 mg. Assuming that this element is easily absorbed from mushrooms by humans, the consumption of approximately 85–150 g of dried or approximately 850–1500 g of fresh fruiting bodies of these three species collected in NW Poland would cover the total demand for this element. Likewise, the entire daily requirement would be covered by 80–140 g of dried *B. edulis* and about 800–1400 g of fresh ones, and in case of *L. scabrum*, by about 90–160 g of dried mushrooms or 900–1600 g of fresh ones, respectively. In the human diet the consumption of 100 g of dried mushrooms (e.g., *B. edulis*) covers 71–100% of the daily requirement for P. The bioavailability of phosphorus is unknown [4,16].

#### 3.2.2. Potassium

Potassium is the major element in mushrooms, along with N and P [9,18,42]. Different species of mushrooms take up similar amounts of potassium [41,42]. Additionally, our research did not show any significant differences in its content in *B. edulis*, *I. badia* and *L. scabrum*; its average concentration was: 7.79 g/kg DM, 4.59 g/kg DM, and 6.91 g/kg DM, respectively (Table 3). The mushrooms we tested were poorer in K, compared to samples collected in other regions of Poland. Potassium content in *L. scabrum* in Poland ranged on average from 21.3 to 52 g/kg DM [42,43,58], in *I. badia* on average from 22.5 to 35.1 g/kg DM [40,42] and *B. edulis* on average from 25–51 g/kg DM [19,39,44,45,59,60]. Higher concentrations in various species of mushrooms were also recorded in other regions of the world, e.g., in Finland from 23.5–26.7 g/kg DM [18], in Japan from 24.9–51.5 g/kg DM [46], in Turkey from 12.6 to 51.0 g/kg DM (in *L. scabrum* 21.1 g/kg DM) [47,61] and in Germany, from 20–40 g/kg DM [4]. The wild mushrooms we analyzed contained less potassium than the cultivated mushrooms *A. bisporus* and *P. ostreatus* [48,49].

The tested mushrooms showed the bioconcentration of K, with the highest BCF coefficient in *L. scabrum* (12.12 in the organic level and 20.32 in the mineral level), (Table 4). This element bioconcentration in mushrooms in the BCF ranging from 2.2 to 93.8 [4,39,40,42,43,53,59]. Whereas Bučinová et al. [54] found a clear influence of the nature of the substrate (organic, mineral) on the value of bioconcentration factor.

Mushrooms contain similar amounts of K as plants. The potassium content in plants, depending on the species, ranges from 2 to 18 g/kg DM [62]. The adequate intake (AI) of K according to WHO [63], EFSA [64] and the Institute of Nutrition and Food [57] ranges from 2400 to 3500 mg/day. The daily requirement for K would be provided by about 370–540 g of dried, or 3700–5400 g of fresh mushrooms from NW Poland, assuming that the accumulated potassium is fully absorbed by the human body. Out of the three tested species, boletuses are the best source of potassium, capable to cover the daily requirement by respectively 308 g to 449 g of dried, or from 3080 g to 4490 g of fresh fruiting bodies. In the human diet the consumption of 100 g of dried mushrooms (e.g., *B. edulis*) covers 22–33% of the daily requirement for K. The bioavailability of K is unknown [4,16]. Mushrooms can enrich a human diet with potassium, but are unlikely to satisfy the full daily requirement.

#### 3.2.3. Magnesium

The tested mushrooms contained significantly less Mg than P and K. A similar distribution of these elements in mushrooms was found by Mattila et al. [16] and Nikkarinen and Mertanen [18]. The mushroom fruiting bodies we collected generally accumulated Mg to a similar degree. Nevertheless, *B. edulis* contained on average significantly more of this element than *I. badia* and birch boletes: 1.37 g/kg DM, 0.91 g/kg DM and 0.89 g/kg DM, respectively (Table 3). The reason for the differences was the greater abundance of absorbable Mg in the substrate on which the mushroom fruiting bodies grew in the Drawa Plain (Table 1). According to Vogt and Edmonds [41] and Rudawska and Leski [42], individual species of mushrooms do not differ significantly in their Mg content. In other regions of Poland, the concentration of Mg in *L. scabrum*, *I. badia* and *B. edulis* ranged from 0.162 g/kg to 1.20 g/kg DM [19,39,40,42,43,44,45,58,59,60]. However, in France the levels found in these mushrooms were from 0.449 to 1.150 g/kg [65], in Finland from 0.696–1.053 g/kg DM [18], in Greece 0.782 g/kg DM [9], in Turkey from 0.850 to 4.54 g/kg DM [47,61] and in Germany from 0.8 to 1, 8 g/kg DM [4]. In Japan, the content of Mg in various species of mushrooms ranged from 0.682 to 1.400 g/kg DM [46]. The mushrooms we analyzed contained amounts of magnesium that were within the range found in cultivated mushrooms: *A. bisporus* and *P. ostreatus* [48,49]. Among the mushrooms we tested, a clear accumulation of Mg was found only in bay boletes, while in the case of *B. edulis* and *L. scabrum*, the bioconcentration factors (BCF) > 1 only in relation to the mineral level (Table 4). Magnesium is generally bioconcentrated in mushrooms in the BCF range from 1.5 to 7.2 [39,40,42,43,53,59]. It happens, however, that under certain environmental conditions, no bioconcentration of this element is observed [4,40]—only in a few cases. Whereas Bučinová et al. [54] found bioconcentration of Mg with mineral horizons, she did not find it with organic horizons of the soil. The content of magnesium in plants ranges from 3 to 10 g/kg [62,64], which is more than in the tested mushrooms (Table 3). The recommended daily allowance (RDA) of Mg according to the Institute of Nutrition and Food [57] ranges from 240 to 420 mg per day. Although mushrooms are not a better source of this macroelement than plants, they can also affect the level of Mg in the human body to some extent. The daily requirement for Mg is provided by about 300 g of the tested dried mushrooms or 3000 g of fresh mushrooms, respectively. In the human diet the consumption of 100 g of dried mushrooms (e.g., *B. edulis*) covers 33–57% of the daily requirement for Mg. The bioavailability of Mg is unknown [4,16].

#### 3.2.4. Calcium

Among the elements tested in mushrooms, calcium was present only in insignificant amounts, showing no significant differences in levels (Table 3). *B. edulis* contained on average 0.12 g/kg, *I. badia* 0.14 g/kg, and *L. scabrum* 0.16 g/kg DM (Table 3). Vogt and Edmonds [41] and Rudawska and Leski [42] draw attention to the lack of differentiation in Ca content in mushrooms, and its small amounts were noticed, among others, by Mattila et al. [16], Nikkarinen and Mertanen [18], Falandysz et al. [19,58], Frankowska et al. [39], Malinowska et al. [40], Zhang et al. [59], Kojta and Falandysz [60]. However, there is a lot of calcium (more than K, P, Mg and Na) in mushrooms from India [1]. The mushrooms we analyzed contained amounts of calcium that were within the range found in cultivated mushrooms: *A. bisporus* and *P. ostreatus* [48,49].

In various regions of Poland, *L. scabrum*, *I. badia* and *B. edulis* contained Ca on average in amounts ranging from 0.018 g/kg to 0.9 g/kg [19,37,39,40,42,43,44,45,58,59,60]. In France, these mushrooms contained from 0.237 to 1.59 g/kg [65], in Finland from 0.041–0.097 g/kg DM [18], in Germany from 0.1 to 0.5 g/kg [47], and in Turkey from 0.0556 to 8.80 g/kg DM [61]. In Japan, the Ca content in various species of mushrooms ranged from 0.10 to 0.625 g/kg DM [46].

The examined *B. edulis*, *I. badia* and *L. scabrum* did not bioconcentration Ca (Table 4). This is consistent with the results obtained by Kalač [4], Chudzyńskii i Falandysz [15], Frankowska et al. [39], Kowalewska et al. [43], Kojta et al. [44] and Zhang et al. [59]. On the other hand, Rudawska and Leski [42] and Falandysz et al. [53] found the bioconcentration of this element in mushrooms. Whereas Bučinová et al. [54] found bioconcentration of Ca in only some fungal species.

The content of calcium in plants ranges from 3 g/kg to 18 g/kg DM [45], therefore mushrooms contain much smaller amounts of Ca than plants (Table 3). The recommended daily allowance (RDA) of Ca according to the Institute of Nutrition and Food [57] ranges from 1000 to 1300 mg. The daily requirement for Ca, if this element was fully available to the human body from mushrooms, would require as much as 9 kg of dried mushrooms or 90 kg of fresh fruiting bodies. In the human diet the consumption of 100 g of dried mushrooms (e.g., *B. edulis*) covers 0.9–1.2% of the daily requirement for Ca.

This means that mushrooms are definitely not a good source of calcium.

#### 3.2.5. Sodium

Sodium was the fourth element in terms of the concentrations of macronutrients found in the tested mushrooms (Table 3). Similar results for *B. edulis* were obtained by Zhang et al. [59] and Nikkarinen and Mertanen [18]. The *B. edulis*, *I. badia* and *L. scabrum* we tested did not differ significantly in the Na content and, respectively, they contained on average: 0.68 g/kg MD, 0.57 g/kg MD, and 0.53 g/kg MD of this element (Table 3). In Poland, the Na content in these species ranged on average from 0.010 to 0.773 g/kg MD [19,37,39,43,45,58,59,60]. On the other hand, in Japan, in various species of mushrooms, the Na content ranged from 0.167 to 1.782 g/kg [46], in Turkey from 0.03 to 4.85 g/kg [47,61], in Finland from 0.065–0.519 g/kg DM [18], and in Germany from 0.1 to 0,8 g/kg [4]. The wild mushrooms we tested had similar amounts of sodium to the cultivated mushrooms *A. bisporus* but more than double that of *P. ostreatus* [48].

The results of our research confirm that mushrooms can bioconcentration Na, with the bioconcentration coefficient being the highest in *B. edulis* and the lowest in *L. scabrum* (Table 4). The properties of mushrooms to bioconcentration Na in mushroom fruiting bodies were previously found by Chudzyński and Falandysz [15], Malinowska et al. [40], Kowalewska et al. [43] and Falandysz et al. [53]; however, this was not confirmed by Kalač [4], Frankowska et al. [39] and Zhang et al. [59].

The content of sodium in plants ranges from 0.3 g/kg to 1.0 g/kg DM [62]. The adequate intake (AI) of Na according to Institute of Nutrition and Food [57] ranges from 1300 to 1500 mg/day. The daily requirement for Na would be provided by about 2 kg of dried mushrooms or 20 kg of fresh mushrooms. In the human diet the consumption of 100 g of dried mushrooms (e.g., *B. edulis*) covers 4–5% of the daily requirement for Na. Thus, in terms of sodium content, mushrooms can only slightly supplement the diet.

### 3.3. The Principal Component Analysis (PCA) for Soil and Mushroom Chemical Composition and Ward’s Cluster Analysis for Macronutrients Content in Soils and Mushrooms

**Figure 1:**
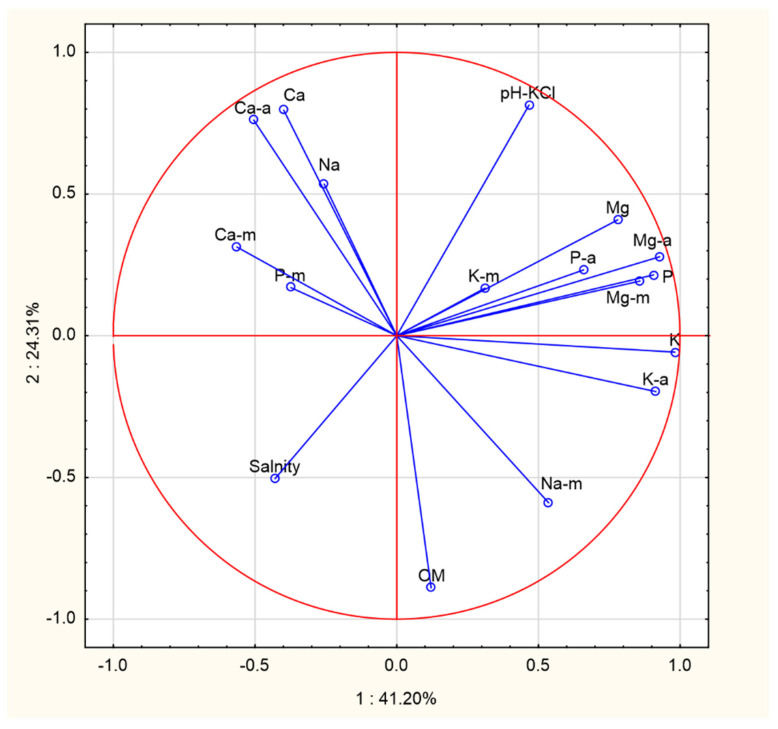
The principal component analysis (PCA) for soil and mushroom chemical composition (K-m—mushrooms, K-a—available, K—total, OM—organic matter).

A higher proportion of organic matter did not significantly affect the content of available and total forms of phosphorus, potassium and magnesium in the soil. On the other hand, the content of available and total forms of calcium and sodium decreased. The lack of correlation between organic matter and the content of macroelements in soils results from its different degree of distribution and negative correlation with pH values. In contrast, a positive correlation was found between organic matter and soil salinity (Figure 1).

The content of phosphorus in fungi was significantly negatively correlated with organic matter, and to a lesser extent negatively correlated with the amount of available and total phosphorus. In contrast, soil pH had no effect on the content of this element in mushrooms. The amount of phosphorus in mushrooms was positively correlated with the amount of calcium, and negatively correlated with the content of magnesium, potassium, and sodium (Figure 1).

Fungi uptake very high amounts of potassium, generally higher than other elements. Potassium uptake depended positively on the content of exchangeable and total forms of this element in the soil and soil pH. The amount of organic matter in the soil did not affect potassium uptake by fungi. The content of potassium was positively correlated with the content of magnesium in fungi, and negatively correlated with the content of phosphorus and calcium (Figure 1).

The amount of magnesium in fungi was positively correlated with the content of available and total forms of magnesium in the soil, to a lesser extent with soil pH, and negatively correlated with the amount of organic matter and salinity. In mushrooms, magnesium content was positively correlated with potassium content, and negatively correlated with phosphorus and calcium content (Figure 1).

The amounts of sodium in the fungi were negatively correlated with the amount of sodium in the soil and soil pH, and positively correlated with the amount of organic matter. Salt concentration had no effect on uptake of this element. In fungi, sodium did not show a strong relationship with magnesium and potassium, but was significantly negatively correlated with calcium and phosphorus (Figure 1).

The relationship between the concentration of elements in mushrooms and their content in soil was already indicated by Garcia et al. [17], Nikkarinen and Mertanen [18]. However, Chudzyński and Falandysz [15] and Malinowska et al. [40] did not find such a dependence.

It was previously pointed out [66,67] that soil parameters (including pH and organic matter) had only a little effect on the content of certain elements in mushrooms. In the tested fruiting bodies, a great uptake of P and Na was found, although the content of these elements in the substrate was relatively low. In sandy soils, phosphorus is present in small amounts and, together with nitrogen, is a deficient element. In such conditions, mycorrhizal mushrooms intentionally accumulate large amounts of this element in order to exchange it for the products of photosynthesis with trees [24,50,51].

**Figure 2:**
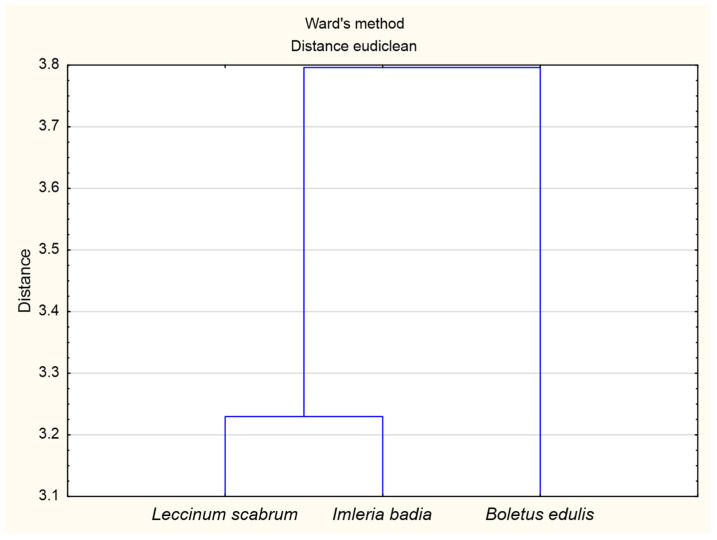
Ward’s cluster analysis for macronutrients content in soils where the mushrooms grew (*L. scabrum*, *I. badia*, *B. edulis*).

**Figure 3:**
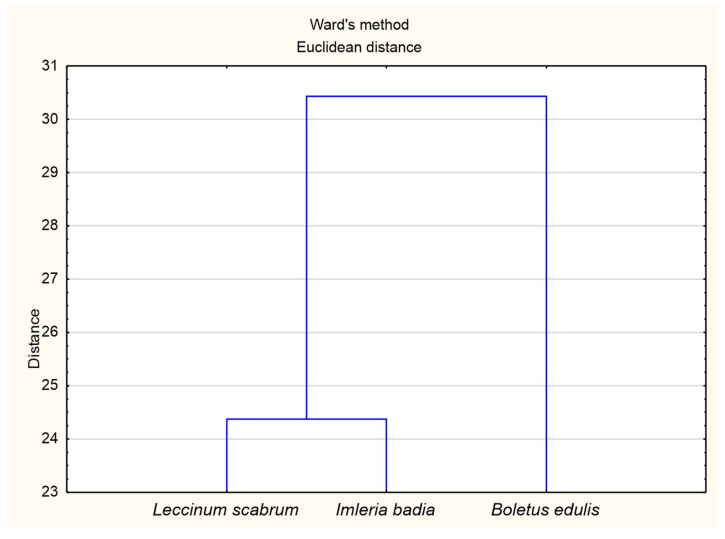
Ward’s cluster analysis for macronutrients content in the mushrooms (*L. scabrum*, *I. badia*, *B. edulis*).

The cluster analysis (Figure 2) identified two groups of substrates under the tested mushrooms that differed in terms of the chemical composition. The first group included the substrates found under boletuses, and the second group contained the substrates under *I. badia* and *L. scabrum*. Similar groups were found in terms of the macronutrient content in mushrooms (1—*B. edulis*, 2—*I. badia* and *L. scabrum*). Our results indicate that *B. edulis* grew on soils with different chemical properties than *I. badia* and *L. scabrum*, which translates into their different chemical composition (Figure 3).

## 4. Conclusions

In NW Poland, mushrooms grew on strongly acidic soils, such as Arenosols and Podzols which showed properties characteristic for these types of soil. The soil under the *B. edulis* differed in the content of macronutrients from the soil under the *I. badia* and *L. scabrum*. The substrates under the *Boletus edulis* we tested were richer in Ca and Mg, but poorer in Na and K, compared to the soils under *B. edulis* in other regions of Poland. *B. edulis*, *I. badia* and *L. scabrum* growing in NW Poland did not differ significantly in the content of P, K, Ca and Na; however, the levels of Mg were significantly higher in *Boletus edulis* growing on the Drawa Plain. The uptake of K, Mg and Ca by the tested mushrooms was positively, and P and Na was negatively correlated with the content of these elements in soil. Only the content of K and Mg in the mushrooms was positively related to soil pH. There was no effect of the amount of organic matter in the soil noticed on the content of macronutrients (except sodium) in mushrooms. Our results indicate that *B. edulis* grow on soils with different chemical properties than *I. badia* and *L. scabrum*, which is reflected in their different chemical composition. The tested fungi bioaccumulated P, K, Mg and Na, while they did not bioaccumulate Ca. P and K bioaccumulated in the greatest amounts, regardless of species. Although the bioaccumulation of K occurred, its concentration in fruiting bodies was lower than in other regions of Poland, which was caused by its lower content in soils. Each of the remaining elements was usually bioaccumulated at a similar level by the fruiting bodies of the species we studied. The exception was the *I. badia*, which bioaccumulated higher amounts of Mg compared to *B. edulis* and *L. scabrum*. The contents of P, K, Ca, and Na were not significantly different between *B. edulis*, bay *I. badia*, and *L. scrabum*. However, *Boletus edulis* was significantly more abundant in Mg. Mushrooms can enrich the human diet with some macronutrients, especially in P, Mg and K. 100 g of dried mushrooms provide almost 100% of the daily requirement for P, about 40% for Mg and about 25% for K. However, the content of Ca and Na in mushrooms is very low and has no significance in the diet.

## Figures and Tables

**Table 1:** Contents of organic matter and available forms of macronutrients, pH and salinity in soils.

Localization	Organic Matter		pH in KCl		Salinity	K	Mg	P	Ca
	%				µS/cm	mg/kg DM			
*B. edulis*									
Soil layer (cm)									
	0–5	5–20	0–5	5–20	5–20	0–5			
UW	46.59 ab	9.17 a	3.25 abc	3.17 ab	132 ab	366.75 a	497.83 ab	1411.67 a	2560.58 ab
DP	67.83 ab	5.06 a	3.54 c	3.39 b	92 a	1082.91 b	982.95 b	2827.94 a	889.38 a
IL	64.38 ab	14.98 a	2.80 ab	3.00 ab	105 a	397.45 a	474.91 a	2834.31 a	1052.81 ab
x	62.05A	9.74A	3.21A	3.19B	109.89A	684.64A	697.55B	2526.73A	1305.86A
*I. badia*									
Soil layer (cm)									
	0–10	10–20	0–10	10–20	10–20	0–10			
UW	59.53 ab	21.24 a	3.19 ac	2.98 ab	117 a	406.46 a	414.42 a	2802.97 a	2831.79 b
DP	75.98 ab	31.54 a	2.65 ab	2.63 ab	277 c	437.53 a	344.93 ab	1338.33 a	1091.92 ab
IL	80.42 a	16.43 a	2.64 b	2.77 a	112 a	467.00 a	351.02 a	1458.74 a	1262.08 ab
x	71.18A	21.37AB	2.87A	2.83A	146.68A	436.89A	375.16A	1972.35A	1855.93A
*L. scabrum*									
Soil layer (cm)									
	0–10	10–20	0–10	10–20	10–20	0–10			
UW	42.06 b	20.80 a	3.25 ac	3.08 ab	128 ab	305.25 a	424.92 a	1250.04 a	2036.09 ab
DP	86.87 a	77.62 b	2.65 ab	2.58 a	245 bc	570.17 ab	378.42 a	1325.87 a	1760.42 ab
IL	79.52 ab	16.86 a	2.53 ab	2.80 ab	128 ab	633.63 ab	388.73 a	1732.64 a	1257.63 ab
x	64.50A	35.22B	2.89A	2.86AB	159.81A	467.06A	402.37A	1402.34A	1748.60A

Abbreviations: UW—Uznam and Wolin. DP—Drawsko Plain. IL—Ińsko Lakeland, x—mean values. Statistical significance of differences between means was determined by ANOVA with Tukey’s post hoc test. The significance was set at *p* < 0.05.

**Table 2:** Total content of macronutrients in soils.

Localization	K		Mg		P		Ca		Na	
	g/kg DM									
*B. edulis*										
Soil layer (cm)										
	0–5	5–20	0–5	5–20	0–5	5–20	0–5	5–20	0–5	5–20
UW	0.54 a	0.24 a	1.57 a	0.31 a	3.16 ab	0.88 ab	3.01 ab	1.18 abc	0.18 a	0.10 a
DP	1.51 b	0.31 a	2.01 a	1.27 b	4.20 b	1.04 ab	1.24 a	0.27 a	0.12 a	0.03 b
IL	0.65 a	0.62 b	1.65 a	0.50 a	3.34 ab	1.350 b	1.19 a	0.37 ab	0.12 a	0.10 a
x	1.00B	0.39A	1.79B	0.69B	3.67B	1.09A	1.60A	0.61A	0.13A	0.08A
*I. badia*										
Soil layer (cm)										
	0–10	10–20	0–10	10–20	0–10	10–20	0–10	10–20	0–10	10–20
UW	0.54 a	0.31 a	1.39 a	0.34 a	3.33 ab	0.86 a	3.53 b	1.08 abc	0.15 a	0.10 a
DP	0.58 a	0.43 ab	1.17 a	0.25 a	2.75 ab	1.26 ab	1.19 ab	0.63 abc	0.12 a	0.09 ab
IL	0.56 a	0.31 a	1.23 a	0.24 a	3.13 b	1.11 ab	1.30 a	0.47 ab	0.10 a	0.08 ab
x	0.56A	0.33A	1.28A	0.28A	3.13AB	1.04A	2.17A	0.75A	0.12A	0.09A
*L. scabrum*										
Soil layer (cm)										
	0–10	10–20	0–10	10–20	0–10	10–20	0–10	10–20	0–10	10–20
UW	0.44 a	0.29 a	1.54 a	0.33 a	2.60 a	0.97 ab	2.38 ab	2.03 c	0.12 a	0.09 ab
DP	0.62 a	0.46 ab	1.13 a	0.31 a	2.67 ab	1.23 ab	2.11 ab	2.33 bc	0.16 a	0.14 a
IL	0.73 ab	0.29 a	1.66 a	0.31 a	3.10 ab	1.01 ab	1.33 ab	0.35 abc	0.12 a	0.10 ab
x	0.57A	0.34A	1.46AB	0.32A	2.76A	1.05A	2.02A	1.66B	0.13A	0.11A

Abbreviations: UW—Uznam and Wolin. DP—Drawsko Plain. IL—Ińsko Lakeland, x—mean values. Statistical significance of differences between means was determined by ANOVA with Tukey’s post hoc test. The significance was set at *p* < 0.05.

**Table 3:** The content of macronutrients in fungi.

Localization	P	K	Mg	Ca	Na
	g/kg DM				
*B. edulis*					
UW	9.96 a	9.63 a	0.97 a	0.14 ab	0.41 a
DP	6.98 a	9.61 a	2.20 b	0.11 a	0.89 a
IL	10.00 a	3.40 a	0.85 a	0.11 a	0.75 a
x	8.92A	7.79A	1.37B	0.12A	0.68A
*I. badia*					
UW	8.16 a	6.04 a	0.99 a	0.13 ab	0.47 a
DP	8.38 a	9.49 a	0.94 a	0.14 ab	0.70 a
IL	8.12 a	0.69 a	0.76 a	0.14 ab	0.61 a
x	8.19A	4.59A	0.89A	0.14A	0.57A
*L. scabrum*					
UW	7.50 a	5.40 a	0.95 a	0.20 b	0.34 a
DP	8.08 a	4.21 a	1.05 a	0.14 ab	0.89 a
IL	7.66 a	12.14 a	0.68 a	0.10 a	0.49 a
x	7.70A	6.91A	0.91A	0.16A	0.53A

Abbreviations: UW—Uznam and Wolin. DP—Drawsko Plain. IL—Ińsko Lakeland, x—mean values. Statistical significance of differences between means was determined by ANOVA with Tukey’s post hoc test. The significance was set at *p* < 0.05.

**Table 4:** Bioconcentration factors of macronutrients in fungi.

Mushroom Species	K		Mg		P		Ca		Na	
*B. edulis*	Soil layer (cm)									
	0–5	5–20	0–5	5–20	0–5	5–20	0–5	5–20	0–5	5–20
	7.79	19.97	0.77	1.99	2.43	8.18	0.08	0.20	5.23	8.50
*I. badia*	Soil layer (cm)									
	0–10	10–20	0–10	10–20	0–10	10–20	0–10	10–20	0–10	10–20
	8.20	2.70	3.59	3.18	2.62	7.88	0.06	0.19	4.75	6.33
*L. scabrum*	Soil layer (cm)									
	0–10	10–20	0–10	10–20	0–10	10–20	0–10	10–20	0–10	10–20
	12.12	20.32	0.62	2.84	2.79	7.33	0.08	0.10	4.08	4.82

## Data Availability

The data presented in this study are available on request from the corresponding author.
